# The SWPER index for women's empowerment in Africa: development and validation of an index based on survey data

**DOI:** 10.1016/S2214-109X(17)30292-9

**Published:** 2017-07-26

**Authors:** Fernanda Ewerling, John W Lynch, Cesar G Victora, Anouka van Eerdewijk, Marcelo Tyszler, Aluisio J D Barros

**Affiliations:** a International Center for Equity in Health, Federal University of Pelotas, Pelotas, Brazil; b School of Public Health, University of Adelaide, Adelaide, SA, Australia; c School of Social and Community Medicine, University of Bristol, Bristol, UK; d Royal Tropical Institute, Amsterdam, Netherlands

## Abstract

**Background:**

The Sustainable Development Goals strongly focus on equity. Goal 5 explicitly aims to empower all women and girls, reinforcing the need to have a reliable indicator to track progress. Our objective was to develop a novel women's empowerment indicator from widely available data sources, broadening opportunities for monitoring and research on women's empowerment.

**Methods:**

We used Demographic and Health Survey data from 34 African countries, targeting currently partnered women. We identified items related to women's empowerment present in most surveys, and used principal component analysis to extract the components. We carried out a convergent validation process using coverage of three health interventions as outcomes; and an external validation process by analysing correlations with the Gender Development Index.

**Findings:**

15 items related to women's empowerment were selected. We retained three components (50% of total variation) which, after rotation, were identified as three dimensions of empowerment: attitude to violence, social independence, and decision making. All dimensions had moderate to high correlation with the Gender Development Index. Social independence was associated with higher coverage of maternal and child interventions; attitude to violence and decision making were more consistently associated with the use of modern contraception.

**Interpretation:**

The index, named Survey-based Women's emPowERment index (SWPER), has potential to widen the research on women's empowerment and to give a better estimate of its effect on health interventions and outcomes. It allows within-country and between-country comparison, as well as time trend analysis, which no other survey-based index provides.

**Funding:**

Bill & Melinda Gates Foundation.

## Introduction

With the call to “leave no one behind”, the Sustainable Development Goals (SDGs) were launched last year by the UN. Gender equity has been specified in many of the SDGs, and goal 5 explicitly aims at the achievement of gender equality and the empowerment of all women and girls.

Women's empowerment is a complex concept for which several definitions exist. The World Bank defines empowerment as “the process of enhancing an individual's or group's capacity to make purposive choices and to transform those choices into desired actions and outcomes”.^1^ Empowerment for women only happens when they can envisage a different life and consider themselves able and entitled to make decisions.^2^ It involves the development of a critical consciousness of women's rights and of gendered power relations, and how these can be changed, so that gender inequity can be overcome.^3^ Empowering women and girls is a goal in itself, as well as a promoter of development.^1^

More empowered women are more likely to use modern contraception, have access to antenatal care and skilled birth attendance, and to provide their children with appropriate nutrition.^4^, ^5^, ^6^, ^7^ Over the past 20 years, progress has been made on gender equity, but slowly. Progress has also been uneven, with large differences between and within countries, and in different wealth groups.^8^, ^9^, ^10^, ^11^

Women's empowerment is difficult to measure because of its abstract and comprehensive nature.^12^ There is consensus that empowerment is multidimensional and is expressed at multiple levels, but less agreement on which dimensions and levels matter more. Some indices have been proposed for low-income and middle-income countries using the Demographic and Health Surveys (DHS),^4^, ^5^, ^6^, ^13^, ^14^, ^15^, ^16^, ^17^, ^18^, ^19^, ^20^ which have included an empowerment module since 1999.^3^ These indices are essentially based on the DHS empowerment module, which includes questions on the woman's involvement in household decisions; employment and type of earnings; control over resources; opinion on wife-beating; and personal ownership of a house or land. These indices have three major limitations: first, the weightings used for the items were chosen subjectively; second, they are only applied to married women because most questions related to empowerment are restricted to this group; and third, they were designed for specific countries or small groups of countries, preventing wider comparisons across countries. The DHS country reports also present two empowerment indices, one composed of decision-making questions and another based on the number of reasons for which the woman thinks wife-beating is justified. Again, these indices are subjectively weighted and are not strictly comparable because not all questions are available in all surveys, and different questions are considered in the indices.

**Research in context**

**Evidence before this study**

The most widely used gender equality indicators, such as the Gender Development Index, are calculated at the country level. In order to include gender in equity analyses, especially for low-income and middle-income countries, an indicator of women's empowerment is needed that can be calculated from national surveys. We searched PubMed, POPLINE, and Google using the terms (((“women”[MeSH Terms] OR “woman”[All Fields]) AND (“power (psychology)”[MeSH Terms] OR (“empowerment”[All Fields])). Most of what we found was from the USAID website, where the Demographic and Health Survey reports are published. We selected publications on the development of novel women's empowerment indicators and those that analysed the association between women's empowerment and reproductive, maternal, and child health outcomes. The indicators we found came mainly from reports that based their analyses on specific countries or surveys. The indicators were usually based on information selected and grouped arbitrarily, and weights to items were defined without any clear strategy.

**Added value of this study**

We propose an indicator (SWPER), which encompasses three well recognised domains of women's empowerment (attitude to violence, social independence, and decision making). The SWPER enables within-country and between-country comparisons, as well as time trend analyses for African countries. No other index has these features. Additionally, it can be calculated at the individual level, enabling detailed analyses to be done of empowerment as an outcome or as a determinant of health.

**Implications of all the available evidence**

Our index enables new studies that were not previously possible. The Sustainable Development Goals put equity high on the agenda, strongly emphasising gender equity. The SWPER is a powerful tool for gender analysis in a region where women's empowerment and gender equity are important issues, and most of the available data come from national surveys.

Several group-level indicators have also been proposed, which condense national or regional information and are generally presented at the country level, for example the Gender Gap Index,^21^ the Gender Development Index, and the Gender Inequality Index.^10^ These indices provide rankings of the countries according to the extent to which women have achieved equality with men. However, they do not enable subnational analyses or subgroup comparisons.

Having a specific SDG on this topic reinforces its importance and the need for a cross-cultural standard indicator to track women's empowerment at different levels to guarantee that the most vulnerable groups are not being left behind and to hold governments and policy makers accountable.^22^ Formulating such an index is a great challenge given the different cultures across countries, since a valid cross-country indicator requires the identification of universally recognised measures of women's empowerment.^13^ We aimed to develop an indicator of empowerment on the basis of individual-level DHS data for African countries that enables comparability between countries and over time. We then aimed to assess how countries compared on this indicator, and to assess the validity of the indicator through its association with key maternal and child health indicators and its correlation with another empowerment indicator.

## Methods

We used data from DHS, which are highly comparable and nationally representative. DHS are one of the main publicly available sources of information for low-income and middle-income countries. Given their focus on maternal and reproductive health, these surveys target women aged 15–49 years. We selected Africa because 37 of the 54 African countries have conducted at least one DHS. Some African countries have the lowest levels of gender equality,^10^, ^21^ poverty levels are high, and there is a unique mix of religions and ethnicity. We used the latest survey available for each country. The ethical responsibility for the DHS lies with the institutions that conducted the surveys in each country; we therefore did not require ethics approval for this study.

We identified questions relevant to women's empowerment that were available in most surveys. Those that were not available in most surveys, at least for partnered women, were discarded. We recoded the answers to the selected questions so that a higher value was given to categories considered to indicate greater empowerment. One of the selected items was age at first birth. Since 5–10% of women had no children at the time of the survey, we imputed data for these cases through single hot-deck imputation, clustering women in groups of age at first cohabitation (appendix). Because several of the relevant items were asked only to women in a union, we restricted our analyses to this group.

We excluded three countries (South Africa, Central African Republic, and the Republic of Congo) because their surveys did not include all the selected items. Thus, 34 countries remained, with surveys from 2003 to 2014. The appendix shows the complete list of surveys (p 1).

Initially, we did principal component analysis in each of the 34 surveys and checked the results (components and items' loadings) for consistency across surveys. We analysed scree plots to define the number of components to be retained and applied orthogonal varimax rotation to the retained components. Next, to achieve a common index that would enable assessment of time trends and cross-country comparisons, we used an approach similar to the development of the International Wealth Index,^23^ performing the principal component analysis on a combined dataset to derive a single indicator of empowerment applicable to all countries.

After creating the index, we assessed its external validity through its correlation with the Gender Development Index, a widely used indicator of gender equality that measures the gender gaps in human development achievements in health, education, and income.^10^ The correlation was measured at country level.

More empowered women usually have higher use of health services, and can provide better feeding and care to their children.^4^, ^5^, ^6^, ^16^, ^24^, ^25^ Thus, we assessed the association between our empowerment index and use of modern contraceptives, institutional delivery, and the prevalence of stunting for the women's last born child to evaluate the convergent validity of the index. We estimated these associations using Poisson regression.^26^ We adjusted all analyses for household wealth. We do not imply that wealth causes empowerment, or the reverse. Because the outcomes are all strongly associated with wealth, by adjusting the analyses we aimed to evaluate whether there was an association of empowerment with the three outcomes independent of wealth. We did the analyses using Stata (release 13).

### Role of the funding source

The funder had no role in the data analysis, data interpretation, or writing of the paper. The corresponding author had full access to all the data and had final responsibility for the decision to submit for publication.

## Results

Initially, we identified 23 items as candidates for our analyses; after checking availability and further theoretical assessment some were excluded (appendix p 1). In the end, we considered 15 items relevant for the index (available in 34 of the 37 surveys), of which five were related to the women's opinion on whether wife-beating was justified in specific situations, and three were related to involvement in household decisions. The other items included the frequency of reading a newspaper or magazine, the woman's education and working status in the previous year, differences in education and age between wife and husband, and the woman's age at first cohabitation and at first birth. Table 1 presents the selected items, and how they were coded.

**Table 1:** Items used in the development of the survey-based women's empowerment index

	**Code or unit**
Beating not justified if wife goes out without telling husband	Justified=–1; don't know=0; not justified =1
Beating not justified if wife neglects the children	Justified=–1; don't know=0; not justified=1
Beating not justified if wife argues with husband	Justified=–1; don't know=0; not justified=1
Beating not justified if wife refuses to have sex with husband	Justified=–1; don't know=0; not justified=1
Beating not justified if wife burns the food	Justified=–1; don't know=0; not justified=1
Frequency of reading newspaper or magazine	Not at all=0; <once a week=1; ≥once a week=2
Respondent worked in past 12 months	No=0; in the past year=1; have a job, but on leave past 7 days=2; currently working=2
Woman's education in completed years of schooling	Years
Education difference: woman's minus husband's completed years of schooling	Years
Age difference: woman's age minus husband's age	Years
Age at first cohabitation	Years
Age of woman at first birth*	Years
Who usually decides on respondent's health care	Husband or other alone=–1; joint=0; respondent alone=1
Who usually decides on large household purchases	Husband or other alone=–1; joint=0; respondent alone=1
Who usually decides on visits to family or relatives	Husband or other alone=–1; joint=0; respondent alone=1

* Imputed for women who had not had a child.

Scree plots showed an abrupt flattening of the curve (slower reduction in the eigenvalues) after the third component for 30 of the 34 countries. Thus, we retained three components in all surveys and proceeded with varimax rotation. The results for all surveys were similar regarding the composition of the components extracted and the item loadings in each component. The appendix (p 3) shows clusters of items with loadings of 0·3 or more. In Cameroon, Gabon, and Lesotho the scree plots suggested retaining two components, and in Mali, four. However, considering their first three components, the pattern loadings were similar to the other countries.

The similarity of results warranted a combined analysis of all datasets. We correlated the women's scores based on the pooled dataset (including all countries) with their scores based solely on each country's dataset. Correlations were all at least 0·99 for the first component, at least 0·90 for the second, and at least 0·93 for the third. The three derived components explained 25%, 14%, and 11% of the total variance, respectively, adding up to 50%.

The components extracted from the analysis represent three domains of empowerment (table 2). The first domain was dominated by questions related to the respondent's opinion about whether wife-beating was justified or not in various scenarios. We labelled it “attitude to violence”. The second domain included items related to education, information (frequency of reading newspaper or magazine), and age at first child birth and at first cohabitation. The differences between the woman and her husband in terms of education and age also appeared in this domain, but with lower loadings. We labelled this domain as “social independence”. The third domain comprised questions about involvement in household decisions and, with a lower loading, whether the respondent worked in the past 12 months. We named it “decision making”. These three domains compose our proposed Survey-based Women's emPowERment index (SWPER; pronounced super).

**Table 2:** Principal component analysis factor loadings, based on the combined dataset including all African countries (n=280 209)

	**Attitude to violence**	**Social independence**	**Decision making**
Beating not justified if wife goes out without telling husband	0·4562	−0·0054	−0·0006
Beating not justified if wife neglects the children	0·4671	−0·0193	−0·0380
Beating not justified if wife argues with husband	0·4594	0·0004	0·0066
Beating not justified if wife refuses to have sex with husband	0·4364	−0·0003	0·0229
Beating not justified if wife burns the food	0·4044	−0·0019	−0·0107
Frequency of reading newspaper or magazine	0·0332	0·3258	0·0891
Woman's education in completed years of schooling	0·0715	0·4178	0·1197
Age of woman at first birth	−0·0335	0·5610	−0·0772
Age at first cohabitation	−0·0155	0·5696	−0·0264
Age difference: woman's age minus husband's age	0·0123	0·1933	0·0931
Education difference: woman's minus husband's years of schooling	−0·0171	0·1943	−0·0348
Who usually decides on respondent's health care	0·0057	0·0028	0·5634
Who usually decides on large household purchases	−0·0229	−0·0087	0·5646
Who usually decides on visits to family or relatives	0·0056	−0·0365	0·5423
Respondent worked in past 12 months	−0·0012	−0·0564	0·1698

**Figure 1:**
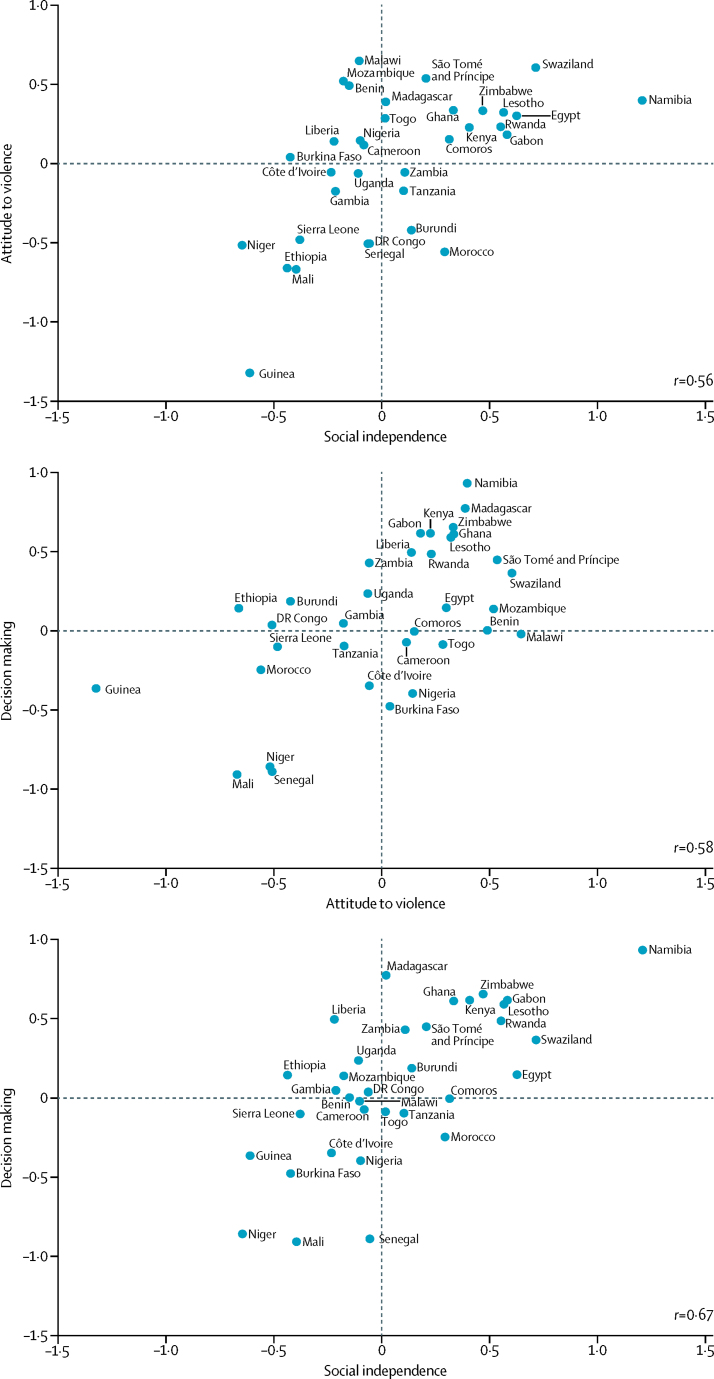
Mean empowerment in each SWPER domain for the 34 African countries analysed Data are centred on zero because SWPER is a standardised measure. Thus, if the average empowerment level is zero, the country empowerment level equals the African average. Positive values indicate that the average empowerment is higher than the African average, and negative values imply the opposite.

Figure 1 shows the average scores for the three SWPER domains plotted against each other. Because the scores are standardised, a zero value means that the country has a score equal to the African average. Negative values imply a worse situation than average; positive values, the opposite. There is a fair degree of correlation between the domains, ranging from 0·56 to 0·67. Countries that appear consistently in the right upper corner of the graphs are the best positioned in terms of empowerment—eg, Namibia and Swaziland. In the left lower corner are countries with the lowest empowerment scores, such as Guinea, Mali, and Senegal. The appendix (p 3) presents a list of the countries, with their empowerment scores and their rankings for the three domains.

The appendix (p 4) also provides the methods to calculate the individual scores for each domain of SWPER. It can be applied to any African country for which all necessary data are available. A Stata do-file with the codes is also available for download.

**Figure 2:**
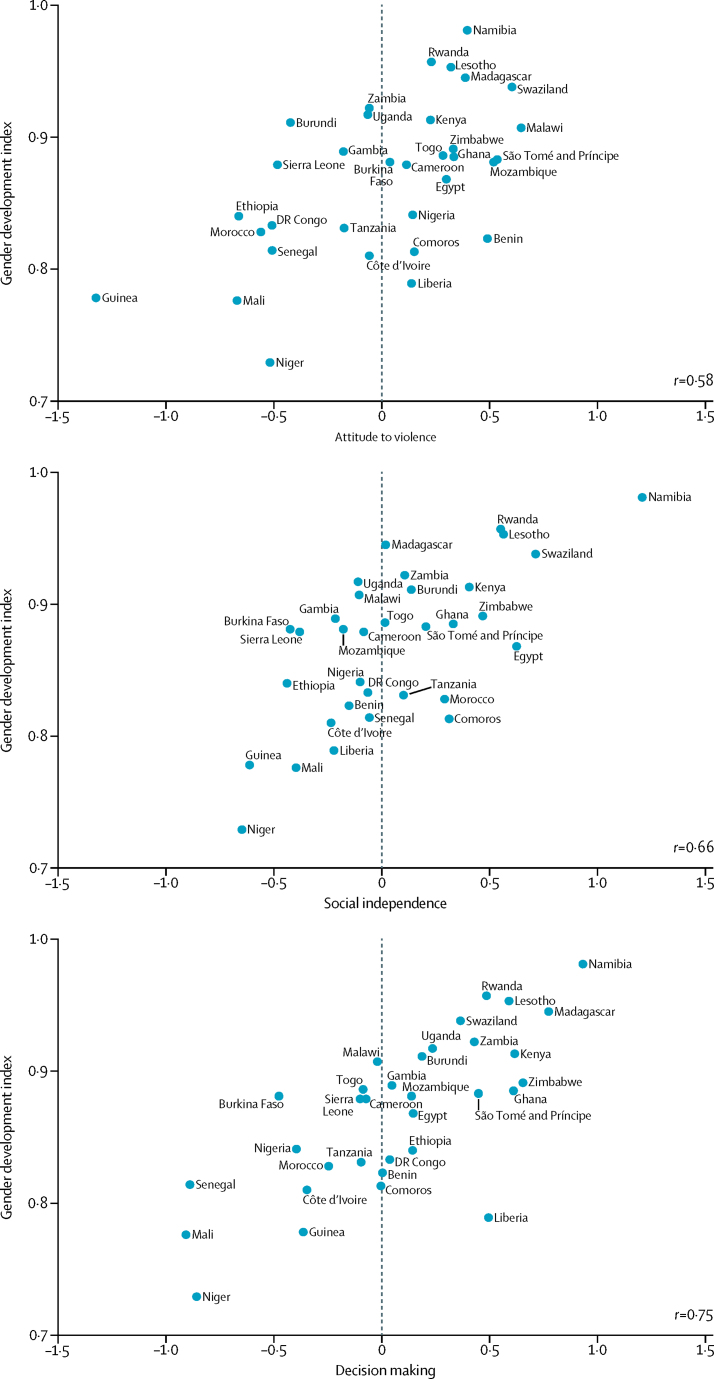
Correlation between the SWPER domains and the Gender Development Index

Figure 2 shows scatter plots of the three SWPER domains against the Gender Development Index. The Gender Development Index is calculated at country level, considering health, education, and command over economic resources. Woman's education is the only common feature with the SWPER. The correlation was 0·75 for decision making, 0·66 for social independence, and 0·58 for attitude to violence.

**Figure 3:**
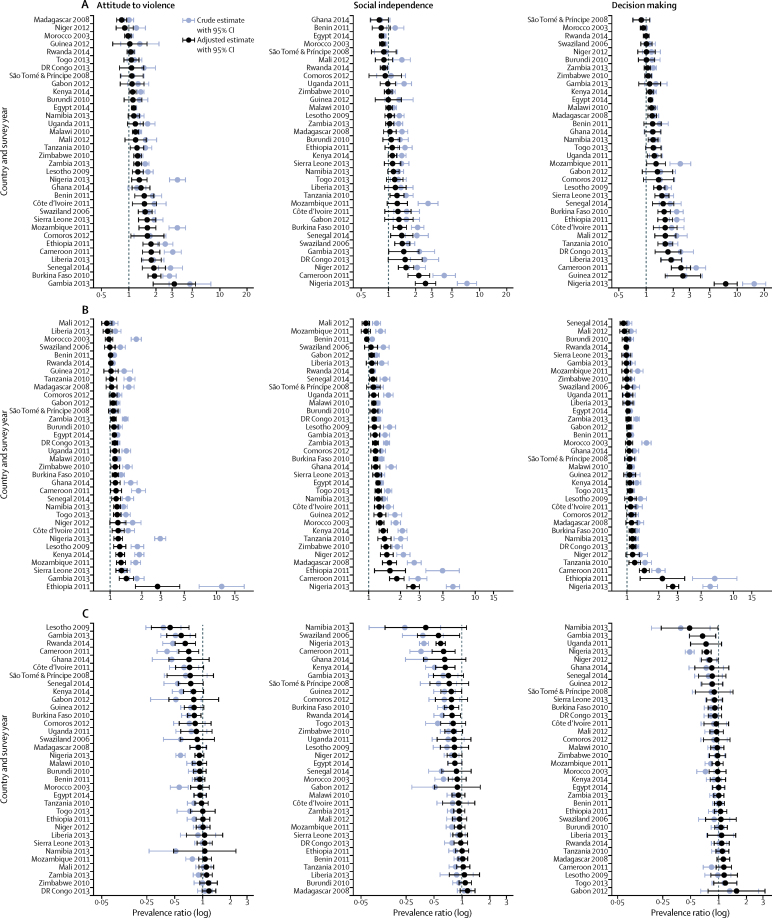
Association between modern contraceptive use (A), institutional delivery (B), stunting prevalence (C), and the SWPER domains Coefficients are prevalence ratios comparing the fifth quintile of empowerment (most empowered) versus the first quintile (least empowered). Crude and household wealth-adjusted results are shown.

We assessed the convergent validity of the SWPER at the individual level, through its association with modern contraceptive use, institutional delivery, and stunting (figure 3). We estimated the prevalence ratio by comparing the coverage in the top quintile of empowerment (Q5) with the bottom quintile (Q1), both crude and adjusted by wealth. Figures are presented in log scale, so that we have symmetry between preventive and risk effects.

Attitude to violence and decision making were more consistently associated with modern contraceptive use than with social independence. 20 of the 34 countries presented positive and statistically significant adjusted effects when we analysed the decision-making domain. In 14 countries, we did not find an association. The strongest association was in Nigeria, where the most empowered women were 8·6 times more likely to use modern contraception.

Social independence was more consistently associated with institutional delivery and stunting than the other domains. 27 of 34 countries had a positive and statistically significant effect with institutional delivery after adjustment by wealth. The strongest effect was again in Nigeria, where the most empowered women were 2·6 times more likely to have had an institutional delivery. Regarding stunting, we found a protective effect of social independence in 25 countries. However, after adjustment, only seven countries showed a statistically significant association, with reductions of up to 44% in stunting prevalence comparing the top and bottom quintiles of empowerment in Swaziland.

## Discussion

The SDGs put gender equity and empowerment of women and girls high on the agenda, reflecting the need to improve opportunities for women^22^ in order to advance social development by closing the gender gap.^27^, ^28^, ^29^ Creating a women's empowerment indicator that can be used in low-income and middle-income countries—where reliable data are scarce—is an important step in this direction.

Although few questions in DHS are related to empowerment, various indicators are based on DHS data.^4^, ^5^, ^6^, ^13^, ^14^, ^15^, ^16^, ^17^, ^18^, ^19^, ^20^ Principal component analysis was used in some of these indicators.^4^, ^18^, ^19^ However, all these measures were proposed to assess women's empowerment in specific contexts and include many items that are not available in all surveys. Our results showed that the data-driven empowerment domains were consistent across African countries, despite contextual differences within the continent. The greatest advantage of SWPER compared with previously proposed indicators is that it can be used across all African countries, allowing comparisons between countries and population subgroups, which are needed to properly analyse equity. It also enables analyses of time trends, which will help track the achievements of women in different subgroups, regions, or countries towards goal 5 of the SDGs.

The correlations between the SWPER domains and the Gender Development Index were high. These results suggest that our index measures relevant aspects of women's empowerment. The SWPER is a useful addition to the Gender Development Index because it can be applied at both the ecological level, as can the Gender Development Index, and at the individual level. Empowerment of women and girls is a goal in itself and promotes development, including economic growth, reduction of poverty, and the accomplishment of human rights.^1^ Empowerment of women and girls could also affect changes in families by providing women with greater autonomy and participation in decision making. We showed that social independence was more consistently related to institutional delivery, and that the attitude to violence and decision-making domains seemed to have a more consistent pattern and greater effect on use of modern contraception. These results accord with studies^4^, ^5^, ^6^ showing that women's empowerment is positively associated with diverse health outcomes and interventions including modern contraceptive use and access to maternal interventions such as antenatal care and skilled birth attendance. Women's empowerment is also associated with the desire for fewer children, although this finding is not consistent across sub-Saharan Africa. This lack of consistency, found in our study and also previous work, could be related to different cultural norms: in some countries, large families are expected by society.^13^ It is a common assumption in multicountry studies that the relation between women's empowerment and outcomes will be different for each setting.^18^

Gender equality also has a central role in children's health.^30^ Findings from a study^16^ done in sub-Saharan Africa and south Asia showed that the more women controlled the economic resources in the household, the more money was spent on their children. Thus, more empowered women would also be more likely to provide their children with appropriate care and nutrition, improving their chances to survive and properly develop.^25^ We found that social independence was more consistently associated with stunting, but the effect disappeared for most countries after adjustment.

We adjusted the associations by wealth to evaluate whether it could explain the associations between empowerment and the three outcomes that are themselves strongly associated with wealth. For use of modern contraception and institutional delivery, the adjusted effect sizes were lower than the crude effect sizes; however, generally they remained statistically significant. Thus, the effects are not explained only by wealth, but also by women's empowerment itself. Further work is needed to assess possible confounders for these associations.

The major limitation of SWPER is that most of the relevant questions were only applied to partnered women. On average, 34% of the women in our dataset were not in a union, ranging from 62% in Namibia (where empowerment levels were high) to only 6% in Egypt. Many empowered women are not necessarily married or will marry later in life. Disabled women and sex workers, who are among the most marginalised and disempowered, might be less likely to be married, and, thus, they are not included in the index. Likewise, our results cannot be generalised to adolescents, many of whom are unmarried. The indicator's scope is also limited by the fact that data from DHS do not cover all aspects of empowerment. For example, they include little on economic and political participation and leadership of women, or on rights to resources and other forms of discrimination against women. Finally, northern and central Africa are to some extent under-represented in our analyses because there were no available data for many countries in these regions.

The age at first birth was considered an important indicator of empowerment, so we included it even though we had to impute data for women who did not have any child by the time of the survey. In most surveys, 5–10% of women had not had a child. These women were generally very young, and did not have enough time to get pregnant after marriage.

Women's empowerment and gender equality might take different forms in different countries across Africa. Yet, the dimensions of empowerment and the correlation structure we identified were very similar across countries, albeit with widely varying scores. The scores from our index must be interpreted in the light of each country's specific context. As a next step, we are exploring the use of this method with surveys in Asia in an attempt to widen the availability of a survey-based empowerment index.

Given the increasing concerns about gender equity, we urge health surveys—one of the main sources of reliable information in low-income and middle-income countries—to incorporate more questions related to empowerment. The DHS already includes a set of questions, but these should be expanded to ensure broader and deeper coverage of issues related to SDG 5. Our proposed empowerment index would also benefit from additional questions on violence against women, social independence, and decision making within the household, to strengthen each of the three key factors. We expect that with the increasing use of these data for estimating women's empowerment, the number of such variables will increase because understanding these issues with maximum representation is crucial. Intervals between DHS are not regular, varying largely across countries. New survey platforms administered more frequently, such as PMA2020, might also include information on women's empowerment, enabling advances towards SDG 5 to be tracked.

SWPER has great potential to widen research on women's empowerment by enabling studies that were not previously possible. The SWPER index enables within-country and between-country comparisons, as well as analysis of time trends, which no other indicator offers. Thus, by improving the comparability of results, we expect SWPER to give a better estimate of the inequalities and its effects of empowerment on maternal, reproductive, and child health.
