# Supplementary material for: The SWPER index for women's empowerment in Africa: development and validation of an index based on survey data
Source: Lancet Glob Health. 2017 Jul 26;5(9):e916–23. doi: 10.1016/S2214-109X(17)30292-9 (PMC5554795; doi:10.1016/S2214-109X(17)30292-9)
Supplement: Supplementary appendix [file mmc1.pdf]

THE LANCET
Global Health

Supplementary appendix

This appendix formed part of the original submission and has been peer reviewed. We post it as supplied by the authors.

Supplement to: Ewerling F, Lynch JW, Victora VG, van Eerdewijk A, Tyszler M, Barros AJD. The SWPER index for women's empowerment in Africa: development and validation of an index based on survey data. *Lancet Glob Health* 2017; published online July 26. http://dx.doi.org/10.1016/S2214-109X(17)30292-9.

The SWPER index for women's empowerment in Africa: development and validation of an index based on survey data

Ewerling, F et al. The Lancet Global Health, 2017.

Supplementary tables and figures

Table S1. Latest DHS surveys from African countries included in the analyses and number of women in the samples.

Country	Year	N	Country	Year	N
1. Benin	2011	11206	18. Malawi	2010	15067
2. Burkina Faso	2010	13192	19. Mali	2012	8737
3. Burundi	2010	5138	20. Morocco	2003	8619
4. Cameroon	2011	9039	21. Mozambique	2011	8643
5. Comoros	2012	2930	22. Namibia	2013	3520
6. Congo DR	2013	12045	23. Niger	2012	9165
7. Côte d'Ivoire	2011	5817	24. Nigeria	2013	26500
8. Egypt	2014	20406	25. Rwanda	2014	6778
9. Ethiopia	2011	10004	26. S Tomé & Príncipe	2008	1715
10. Gabon	2012	4100	27. Senegal	2014	5251
11. Gambia	2013	6423	28. Sierra Leone	2013	10254
12. Ghana	2014	5343	29. Swaziland	2006	1888
13. Guinea	2012	6641	30. Tanzania	2010	6233
14. Kenya	2014	8833	31. Togo	2013	6161
15. Lesotho	2009	4069	32. Uganda	2011	5150
16. Liberia	2013	5708	33. Zambia	2013	9088
17. Madagascar	2008	11102	34. Zimbabwe	2010	5444

Table S2. DHS variables included in the empowerment index and reasons for exclusion.

Variable	Included	Reason for exclusion
Beating justified if wife goes out without telling husband	Yes	
Beating justified if wife neglects the children	Yes	
Beating justified if wife argues with husband	Yes	
Beating justified if wife refuses to have sex with husband	Yes	
Beating justified if wife burns the food	Yes	
Frequency of reading newspaper or magazine	Yes	
Respondent worked in last 12 months	Yes	
Woman's education	Yes	
Education difference: woman's minus husband's years of schooling	Yes	
Age difference: woman's minus husband's age	Yes	
Age at first cohabitation	Yes	
Age of respondent at 1st birth	Yes	
Who usually decides on respondent's health care	Yes	
Who usually decides on large household purchases	Yes	
Who usually decides on visits to family or relatives	Yes	
Who usually decides on daily household purchases	No	Not available in all surveys
Whether she can refuse to have sex with the husband	No	Not available in all surveys
Ever used anything or tried to delay or avoid getting pregnant	No	Not available in all surveys
Type of earnings from respondent's work	No	Asked only to women employed in last year
Can you go to a health centre or hospital alone or with your young children?	No	Not available in all surveys
Decision maker for using contraception	No	Asked only to women using contraception
Ownership of house or land	No	Not available in all surveys
Ownership of a personal mobile phone	No	Usually a household question; personal information is not available in all surveys.

Table S3. Composition patterns of the SWPER domains.

Country	N	Variable numbers*																
		Attitude to violence					Social independence						Decision making					
		1	2	3	4	5	6	7	8	9	10	11	12	13	14	11	6	7
Benin	11206	x	x	x	x	x	x	x	x	x			x	x	x			
Burkina Faso	13192	x	x	x	x	x	x	x	x	x			x	x	x			
Burundi	5138	x	x	x	x	x	x	x	x	x			x	x	x			
Cameroon	9039	x	x	x	x	x	x	x	x	x			x	x	x	x		
Comoros	2930	x	x	x	x	x	x	x	x	x			x	x	x			
Côte d'Ivoire	12045	x	x	x	x	x	x	x	x	x			x	x	x			
Gambia	5817	x	x	x	x	x	x	x	x	x			x	x	x			
Ghana	20406	x	x	x	x	x	x	x	x	x			x	x	x			
Guinea	10004	x	x	x	x	x	x	x	x	x			x	x	x			
Kenya	4100	x	x	x	x	x	x	x	x	x			x	x	x	x		
Madagascar	6423	x	x	x	x	x	x	x	x	x			x	x	x			
Mali	5343	x	x	x	x	x	x	x	x	x			x	x	x			
Niger	6641	x	x	x	x	x	x	x	x	x			x	x	x			
Nigeria	8833	x	x	x	x	x	x	x	x	x			x	x	x	x		
Senegal	4069	x	x	x	x	x	x	x	x	x			x	x	x	x		
Sierra Leone	5708	x	x	x	x	x	x	x	x	x			x	x	x			
Swaziland	11102	x	x	x	x	x	x	x	x	x			x	x	x			
Togo	15067	x	x	x	x	x	x	x	x	x			x	x	x	x		
Uganda	8737	x	x	x	x	x	x	x	x	x			x	x	x			
Zambia	8619	x	x	x	x	x	x	x	x	x			x	x	x			
Zimbabwe	8643	x	x	x	x	x	x	x	x	x			x	x	x			
Congo DR	3520	x	x	x	x	x	x	x	x	x	x		x	x	x			
Gabon	9165	x	x	x	x	x	x	x	x	x	x		x	x	x			
Liberia	26500	x	x	x	x	x	x	x	x	x	x		x	x	x			
S Tomé & Príncipe	6685	x	x	x	x	x	x	x	x	x	x		x	x	x			
Egypt	1715	x	x	x	x	x		x	x	x			x	x	x			
Ethiopia	5251	x	x	x	x	x		x	x	x			x	x	x			
Lesotho	10254	x	x	x	x	x		x	x	x			x	x	x			
Malawi	1888	x	x	x	x	x		x	x	x			x	x	x			
Morocco	6233	x	x	x	x	x		x	x	x			x	x	x			
Rwanda	6778	x	x	x	x	x		x	x	x			x	x	x			
Mozambique	5150	x	x	x	x	x			x	x			x	x	x		x	x
Tanzania	9088	x	x	x	x	x		x	x	x	x		x	x	x			
Namibia	5444	x	x	x	x	x	x	x	x	x		x	x	x	x			

Key to variable numbers: Beating not justified: (1) if woman goes out without telling husband; (2) if woman neglects the children; (3) if woman argues with husband; (4) if woman refuses to have sex with husband; (5) if woman burns the food; (6) Frequency of reading newspaper; (7) Education; (8) Age at 1st birth; (9) Age at 1st cohabitation; (10) Education difference (woman's minus husband's years of schooling); (11) Work; Who usually decides on: (12) respondent's healthcare; (13) large household purchases; (14) visits to family or relatives.

Table S4. Tertiles (green=top; yellow=intermediate; red=bottom) and country rankings of the average national scores of the three domains. Top tertile represent the countries with higher average empowerment levels.

Country	Year	Tertiles			Ranking		
		Attitude to violence	Social Independence	Decision making	Attitude to violence	Social Independence	Decision making
Namibia	2013	3	3	3	6	1	1
Lesotho	2009	3	3	3	10	5	7
Zimbabwe	2010	3	3	3	9	7	3
Ghana	2014	3	3	3	8	9	6
Swaziland	2006	3	3	2	2	2	12
Egypt	2014	3	3	2	11	3	15
Gabon	2012	2	3	3	15	4	4
Rwanda	2014	2	3	3	13	6	9
Kenya	2014	2	3	3	14	8	5
S Tomé & Principe	2008	3	2	3	3	12	10
Madagascar	2008	3	2	3	7	16	2
Comoros	2012	2	3	2	16	10	21
Zambia	2013	2	2	3	22	14	11
Malawi	2010	3	2	2	1	22	22
Benin	2011	3	1	2	5	24	20
Mozambique	2011	3	1	2	4	25	17
Liberia	2013	2	1	3	18	27	8
Morocco	2003	1	3	1	31	11	27
Burundi	2010	1	2	2	26	13	14
Togo	2013	2	2	1	12	17	24
Congo DR	2013	1	2	2	29	19	19
Cameroon	2011	2	2	1	19	20	23
Nigeria	2013	2	2	1	17	21	30
Tanzania	2010	1	2	1	24	15	25
Senegal	2014	1	2	1	28	18	33
Uganda	2011	1	1	2	23	23	13
Gambia	2013	1	1	2	25	26	18
Cote d'Ivoire	2011	2	1	1	21	28	28
Burkina Faso	2010	2	1	1	20	31	31
Ethiopia	2011	1	1	2	32	32	16
Sierra Leone	2013	1	1	1	27	29	26
Mali	2012	1	1	1	33	30	34
Guinea	2012	1	1	1	34	33	29
Niger	2012	1	1	1	30	34	32

How to calculate the SWPER for a specific survey

The equation used to estimate individual standardized scores for each of the PCA *j* components is given by:

$$S_{ij} = \frac{\left[[\lambda_{1j}(x_{1i} - \bar{x}_1)] + [(\lambda_{2j}(x_{2i} - \bar{x}_2)] + \cdots + [\lambda_{15j}(x_{15i} - \bar{x}_{15})]\right]}{\sigma_j} \quad (1)$$

where S_{ij} are the individual standardized scores for individual *i* and component *j*; $x_{1j}, \ldots, x_{15j}$ are the individual values for variables x1-x15 included in the PCA analyses; σ_j are the standard deviations of the predicted scores of each component *j*. The weight given to each of the 15 variables in each component *j* is defined as:

$$\lambda_{vj} = \frac{\varphi_{vj}}{\sigma_v} \quad (2)$$

Where φ_{vj} is the PCA loading for each of the variables v in each domain *j* and σ_v is the standard deviation of each variable v in the combined dataset.
By using simple algebra, we can simplify the equation above to:

$$S_{ij} = \frac{\left[-(\sum_{v=1}^{15} \lambda_{vj}\, \bar{x}_v) + \sum_{v=1}^{15}(\lambda_{vj} x_{vi})\right]}{\sigma_j} \quad (3)$$

Please, follow the next steps to calculate the standardized individual SWPER scores for any African country of your interest[1]:

1. Recode variables

The first step is to recode the variables as it is shown in Table S5, below.

1.1. Imputation of woman's age at first birth

To procced the imputation of age at first birth for nulliparous women, we used single hotdeck imputation. This method randomly selects the value to be imputed for a missing case from a group of individuals that are similar to it in terms of a variable or a group of variables. In this case, women were clustered in groups of age at first cohabitation. This variable was selected because it had the highest correlation with age at first birth, and other variables did not add much predictive power in a regression model. Despite the current preference for multiple imputation, we used a single imputation approach because procedures for principal component analysis with multiple imputation data are not commonly available, and the percentage of missing information was not so high that overall variance would be significantly reduced by use of single imputation.

2. Calculate the individual scores

Using the equations below, it is possible to estimate the scores for the three SWPER domains:

$$Score_{\,Attitude\ to\ violence_i} = \frac{\left[(-0.950) + \sum_{v=1}^{15}(\lambda_{v1} x_{vi})\right]}{1.818}$$

$$Score_{\,Social\ independence_i} = \frac{\left[(-5.360) + \sum_{v=1}^{15}(\lambda_{v2} x_{vi})\right]}{1.475}$$

$$Score_{\,Decision\ making_i} = \frac{\left[0.857 + \sum_{v=1}^{15}(\lambda_{v3} x_{vi})\right]}{1.417}$$

[1] A Stata do-file with all procedures required for the calculation of the SWPER Index scores is available from the Dropbox link <https://www.dropbox.com/sh/6jw57xtp824qsbh/AAC5kOeVBG2o_aBbzdHPCnlYa?dl=0>.

Table S5. Variables used in the development of the survey-based women's empowerment index.

Variable	Code or unit
Beating justified if:<br>1. wife goes out without telling husband<br>2. wife neglects the children<br>3. wife argues with husband<br>4. wife refuses to have sex with husband<br>5. wife burns the food	Yes = -1; DK=0; No=1
6. Frequency of reading newspaper or magazine	Not at all=0;<br><once a week=1;<br>≥once a week=2
7. Respondent worked in last 12 months	No = 0;<br>In the past year = 1;<br>Have a job, but on leave last 7 days = 2;<br>Currently working = 2
8. Woman education in completed years of schooling	Years
9. Education difference: woman's minus husband's years of schooling	Years
10. Age difference: woman's minus husband's age	Years
11. Age at first cohabitation	Years
12. Age of woman at first birth*	Years
Who usually decides on:<br>13. Respondent's health care<br>14. Large household purchases<br>15. Visits to family or relatives	Husband/other alone= -1;<br>joint=0;<br>respondent alone=1

* This variable was imputed for women who had not had a child, please see section 1.1 for details.

Where x_{vi} is the value of variables v for each individual i and $\lambda_{v1} - \lambda_{v3}$ are the variable weights, that can be found in Table S6.

Table S6. Variable weights used in the equations for estimating individual scores for each domain of the SWPER Index.

Variable (v)	λ_{v1} **Attitude to violence**	λ_{v2} **Social independence**	λ_{v3} **Decision-making**
1. Beating not justified if wife goes out without telling husband	0·489	-0·006	-0·001
2. Beating not justified if wife neglects the children	0·493	-0·020	-0·040
3. Beating not justified if wife argues with husband	0·501	0·000	0·007
4. Beating not justified if wife refuses to have sex with husband	0·493	0·000	0·026
5. Beating not justified if wife burns the food	0·546	-0·003	-0·014
6. Frequency of reading newspaper or magazine	0·056	0·549	0·150
7. Woman education	0·015	0·090	0·026
8. Age of respondent at first birth	-0·008	0·141	-0·019
9. Age at first cohabitation	-0·004	0·131	-0·006
10. Age difference: woman's minus husband's age	0·002	0·026	0·012
11. Education difference: woman's minus husband's years of schooling	-0·004	0·050	-0·009
12. Who usually decides on respondent's health care	0·008	0·004	0·770
13. Who usually decides on large household purchases	-0·034	-0·013	0·831
14. Who usually decides on visits to family or relatives	0·008	-0·052	0·768
15. Respondent worked in last 12 months	-0·001	-0·060	0·180
